# Evaluation of AI for prostate cancer detection in biparametric-MRI screening population data

**DOI:** 10.1007/s00330-025-12198-5

**Published:** 2025-12-08

**Authors:** Fredrik Langkilde, Magnus Gren, Jonas Wallström, Stefan Kuczera, Stephan E. Maier

**Affiliations:** 1 Department of Radiology, Institute of Clinical Sciences, Sahlgrenska Academy, University of Gothenburg, Gothenburg, Sweden; 2 Department of Radiology, Sahlgrenska University Hospital, Gothenburg, Sweden; 3 Department of Radiology, Brigham and Women’s Hospital, Harvard Medical School, Boston, MA USA

**Keywords:** Screening, Artificial intelligence, Prostate

## Abstract

**Objective:**

The goal of this study was to curate a prostate MRI dataset from a screening population and to train and evaluate a deep-learning segmentation method on the same data.

**Materials and methods:**

An artificial intelligence (AI) system, based on a deep-learning-based segmentation model (nnU-Net method), was trained and evaluated with MRI data from a prostate cancer screening population (G2-trial). The goal of the AI was to detect clinically significant prostate cancer (csPC), defined as International Society of Urological Pathology (ISUP) grade 2 or higher. The AI system was compared to the performance of radiologists using PI-RADS v2 evaluation metrics. Histopathology was used as the reference standard in the dataset. To better verify negative cases, 288 men were subject to systematic biopsies regardless of MRI findings, and all men had at least 3 years of follow-up.

**Results:**

A total of 1354 MRI examinations in 1254 men with a median age of 58 years (range 50–63 years) were randomly divided into a training set (1086 examinations) and a test set (268 examinations). The resulting area under the receiver operating characteristic curve (AUROC) was 0.83 (95% CI 0.73–0.92) for the AI system; however, with significantly lower specificity at matched sensitivity levels compared to radiologists.

**Conclusion:**

A prostate MRI dataset from a screening population with histological confirmation was curated and evaluated with AI. The neural network trained and tested on this data produced lower specificities than the radiologists.

**Key Points:**

***Question*** *Does an AI system trained in a screening cohort perform as well as radiologists?*

***Findings*** *An AI trained on screening data achieved an AUROC of 0.83 (95% CI 0.73–0.92) with lower specificity at the same sensitivity levels as radiologists.*

***Clinical relevance*** *An AI system trained in a screening population has lower specificity than radiologists using PI-RADS v2.*

**Graphical Abstract:**

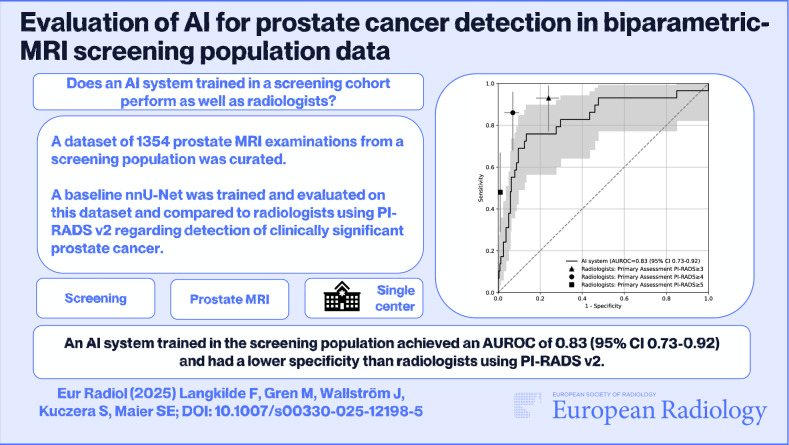

## Introduction

In Europe, MRI is recommended before biopsies as a part of the diagnostic pathway for prostate cancer (PC) [1]. The benefits of adding MRI are a decrease in the number of biopsies needed [2], enabling MRI-targeted biopsies [3, 4], and reduced overdiagnosis [5]. If a screening program is to be introduced, efforts must be made to maximize the efficiency of the screening program to make it cost-effective and feasible. Radiological interpretation of MRI examinations is costly, and there is a shortage of radiologists with that competence. Artificial intelligence (AI)-assisted workflows can potentially reduce the time needed for image interpretation and reduce costs. There are also some issues that are more prominent in screening that might be aided by AI-assisted interpretation, such as inter-reader and inter-center variability and the automatic rule-out of negative examinations that might not require interpretation by a radiologist at all [6]. However, evidence confirming these benefits is lacking [7–9]. A multitude of commercial AI solutions are available, but there is little transparency on what kind of training data was used, and there are few studies on the clinical efficacy of these programs [10]. Programs trained and evaluated exclusively on clinical data with a higher prevalence of PC than a screening population may have unforeseen effects when used in screening populations, such as overdiagnosis, which could reduce the benefits of using MRI in the first place.

This study aimed to curate a dataset from a prostate cancer screening population and to train and evaluate a neural network with it using the baseline version of the deep-learning-based segmentation method nnU-Net [11].

## Materials and methods

### Ethical considerations

The present study was approved by the Swedish Ethical Review Authority under the registration number 2019-04453.

### Study population

The study population consisted of men retrospectively selected from the Göteborg Prostate Cancer Screening 2 Trial (G2-trial) (IS-RCTN94604465). The G2-trial is described in detail elsewhere [12], but in short, men aged 50–60 years were randomized to either a control group or a screening group. All men in the screening group were invited for prostate-specific antigen (PSA)-testing and then further randomized into one of three arms: (1) a PSA ≥ 3 ng/mL, with prostate MRI and systematic biopsy regardless of MRI result, targeted biopsies added in case of MRI findings with PI-RADS ≥ 3, (2) a PSA ≥ 3 ng/mL with prostate MRI and targeted biopsies added in case of MRI findings with PI-RADS ≥ 3 and (3) same as (2) but a PSA ≥ 1.8 ng/mL. Re-invitation intervals varied depending on PSA level, MRI results, and biopsy results. The present study includes all men who underwent an MRI examination of the prostate under the G2-trial between October 25th, 2015, and March 2nd, 2019. Exclusion criteria were incomplete biparametric MRI data or excessive artifacts on any of the scans.

### Follow-up

Data from the G2-trial study database and the Swedish Cancer Registry were used to determine whether a patient had PC during the follow-up period. The data were extracted from both databases on January 31, 2023.

### MRI data

All examinations were performed on a Philips Achieva dStream 3-T MRI scanner at Sahlgrenska University Hospital using a pelvic-phased array coil for signal reception. A multiparametric MRI protocol was used. The axial T_2_-weighted (T2W) and diffusion-weighted imaging (DWI) scans were acquired in the same oblique axial plane along the prostate base. Scan parameters for the oblique axial T2W scan were as follows: TR, 3906 ms; TE, 105 ms; FOV, 180 × 180 mm; acquisition matrix size, 200 × 212; and slice thickness, 1.5 mm. The oblique axial diffusion-weighted imaging (DWI) scan used the following scan parameters: TR, 4000 ms; TE, 78 ms; FOV, 280 × 233 mm; acquisition matrix size, 92 × 77; slice thickness, 3 mm; and 2-fold parallel coil acceleration. The DWI scan included the b-values 0, 100, 1000, and 1500 s/mm^2^, with 6-fold signal averaging, and the geometric mean of three orthogonal encoding directions for all non-zero b-values. The apparent diffusion coefficient (ADC) map was calculated by least square fitting of the logarithmized signals using all b-values. The above-mentioned T2W scan, the DWI with *b*-value 1500 s/mm^2^, and the ADC map were used as input to the neural network. In addition, T2W scans with 3 mm slice thickness in three orthogonal planes and an oblique axial dynamic contrast-enhanced (DCE) scan were obtained but only used for the primary MRI assessment and not as input to the neural network.

### Determination of the reference standard on MRI examinations

**Fig. 1:**
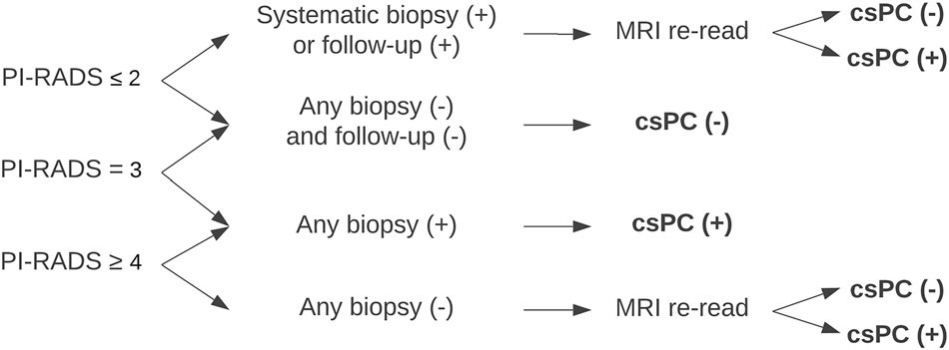
Flowchart for the determination of the reference standard. All PI-RADS scores are the biparametric scores from the primary MRI assessment. (+) for biopsies and follow-up indicates findings of ISUP-grade 2 or higher. “Any biopsy” indicates either a systematic or targeted biopsy. For a biopsy to be positive (+), the sector of the MRI lesion and the biopsy had to match. Systematic biopsies could only upgrade findings from targeted biopsies. csPC(+) and csPC(−) indicate the presence of clinically significant prostate cancer (csPC), respectively, no prostate cancer. One out of two radiologists performed MRI re-read with all available data from the follow-up. If the presence of csPC could be confirmed, it was added

Determination of the reference standard was similar to the strategy used by Saha et el [13]. A flow chart illustrating the selection of the reference standard is provided in Fig. 1. Three board-certified radiologists with over 5 years of experience in diagnostic evaluation of prostate MRI read the MRI exams. For each MRI exam, two out of these three readers performed an initial read in consensus (primary MRI assessment). Multiparametric MRI data were used, and findings were reported using PI-RADS v2 [14]. Both a multiparametric and a biparametric PI-RADS score (without DCE series) were reported for the primary MRI assessment. The multiparametric MRI score guided the biopsy procedure according to the G2-trial study protocol [12]; meanwhile, the biparametric score was used for comparison with the AI system. The International Society of Urological Pathology (ISUP) grading system was used for histology grading [15]. Clinically significant prostate cancer (csPC) was defined as ISUP grade ≥ 2. Negative biopsies were defined as benign or ISUP grade 1 or lower.

All MRI findings with a biparametric PI-RADS score ≥ 3 and a following biopsy showing csPC from the same sector were considered csPC. All MRI examinations with a biparametric PI-RADS score ≤ 3, negative biopsies, and no PC diagnosis during the follow-up period were categorized as ‘no csPC.’ All other MRI examinations were re-read by at least one of two radiologists, who had access to all available data from the follow-up period. The MRI examinations with biparametric PI-RADS ≤ 2 and a csPC diagnosis during the follow-up period (either found in systematic biopsies within the study or biopsies outside the study) were re-read to determine if csPC was missed on the MRI examination. The MRI examinations with biparametric PI-RADS ≥ 4 and no csPC diagnosis during the follow-up period were re-read to determine if csPC was missed on biopsies. If the radiologists doing the re-read deemed the presence of csPC to be likely, it was considered csPC, despite no confirmed histological diagnosis of csPC. Otherwise, it was categorized as ‘no csPC.’ If systematic biopsies were performed in addition to MRI-targeted biopsies, the ISUP grade could only be upgraded. If a prostatectomy was performed, subsequent whole-gland histology findings superseded prior biopsy findings.

One radiologist, with over 5 years of prostate MRI experience, segmented all csPC lesions using the software ITK-snap (version 4.0.1) [16]. If the lesion was hard to delineate, a second radiologist with approximately 10 years of prostate MRI experience was consulted. All available information for that case was used to determine the lesion’s location and extent, e.g., primary MRI reports, sectors for biopsy, and prostatectomy findings.

### MRI data preparation

The curated MRI data were randomly divided into a set to be used only for training (80% of all cases) and another set to be used only for testing (20% of all cases). No consideration to follow-up visits was taken during this randomization. All diffusion-weighted images and the ADC map were transformed to the same in-plane spatial resolution and number of slices as the T2W images using linear interpolation with TorchIO (version 0.19.2) [17]. Rigid registration was performed using Elastix (version 5.1.0) [18] with the transformed b0-image as moving image and the T2W image as fixed. The same registration was subsequently applied to the transformed high b-value image and the ADC map. The code used for executing these procedures and two example cases can be found on our GitHub; please see the supplementary material. The axial T2W images, the b = 1500 s/mm^2^ diffusion-weighted images, and the ADC maps were used as input for the neural network.

### Training of the neural network and inference on the test set

The deep-learning-based segmentation method nnU-Net (version 2.5.1) was used [11]. For a detailed explanation of how nnU-Net v2 operates, the original publication and supplementary information can be consulted [11]. The built-in experiment planning and preprocessing were used. The 2D and 3D full resolution configurations were trained; the 3D low resolution and 3D cascade were omitted due to the relatively low resolution of the input images (interpolated to 432 × 432). Default five-fold cross-validation was used, and nnU-Net identified the single best one or an ensemble of the two configurations based on the Dice score in the validation set. Ensembling was done by averaging softmax probabilities. Five Nvidia A100 GPUs with 80 GB of VRAM were used for training. After training, inference was conducted on the test set using the best configuration. Detection maps were obtained by saving softmax predictions and extracting lesion candidates from these predictions using the report-guided annotation (RGA) tool with dynamic lesion extraction settings as described in detail in ref. [19]. The code used for executing these procedures and two examples can be found on our GitHub; please see the supplementary material. The RGA takes the highest voxel value from the softmax prediction and all connected voxels in 3D with a value of at least 40% of the highest voxel value, creating one lesion candidate. All of the voxels included in the first lesion and the connected voxels are then removed, and the procedure is repeated to find additional lesion candidates. All voxels in the same lesion candidate are assigned the value of the highest voxel in the lesion. For the statistical analysis, the highest softmax value for each case was used as a likelihood score (0 to 1) in the final evaluation.

### Statistical analysis

The diagnostic performance of the AI system and the radiologists’ primary MRI assessment using PI-RADS v2 on biparametric data was evaluated according to their case-level predictions for csPC. The receiver operating curve (ROC) was determined, and the corresponding area under the curve (AUROC) was calculated. Moreover, the specificity and sensitivity for the radiologists’ primary assessment at PI-RADS 3, 4, and 5 were determined and compared with the mean specificity of the AI. The 95% confidence intervals were calculated for all of the above measurements. For AUROC, DeLong’s method was used [20], for sensitivities and specificities of the radiologists and for the ROC curve, Clopper-Pearson’s method was used, and for the mean difference in specificity, bootstrapping was applied to calculate the confidence intervals. Bootstrapping was non-parametric with replacement and 10,000 resamples, and confidence intervals were calculated based on the percentile method. If the confidence interval of the mean difference did not overlap zero, it was considered significantly different. Statistical analyses were performed in R (version 4.4.1), and the script is available on our GitHub; please see the supplementary material.

## Results

### Final dataset

**Fig. 2:**
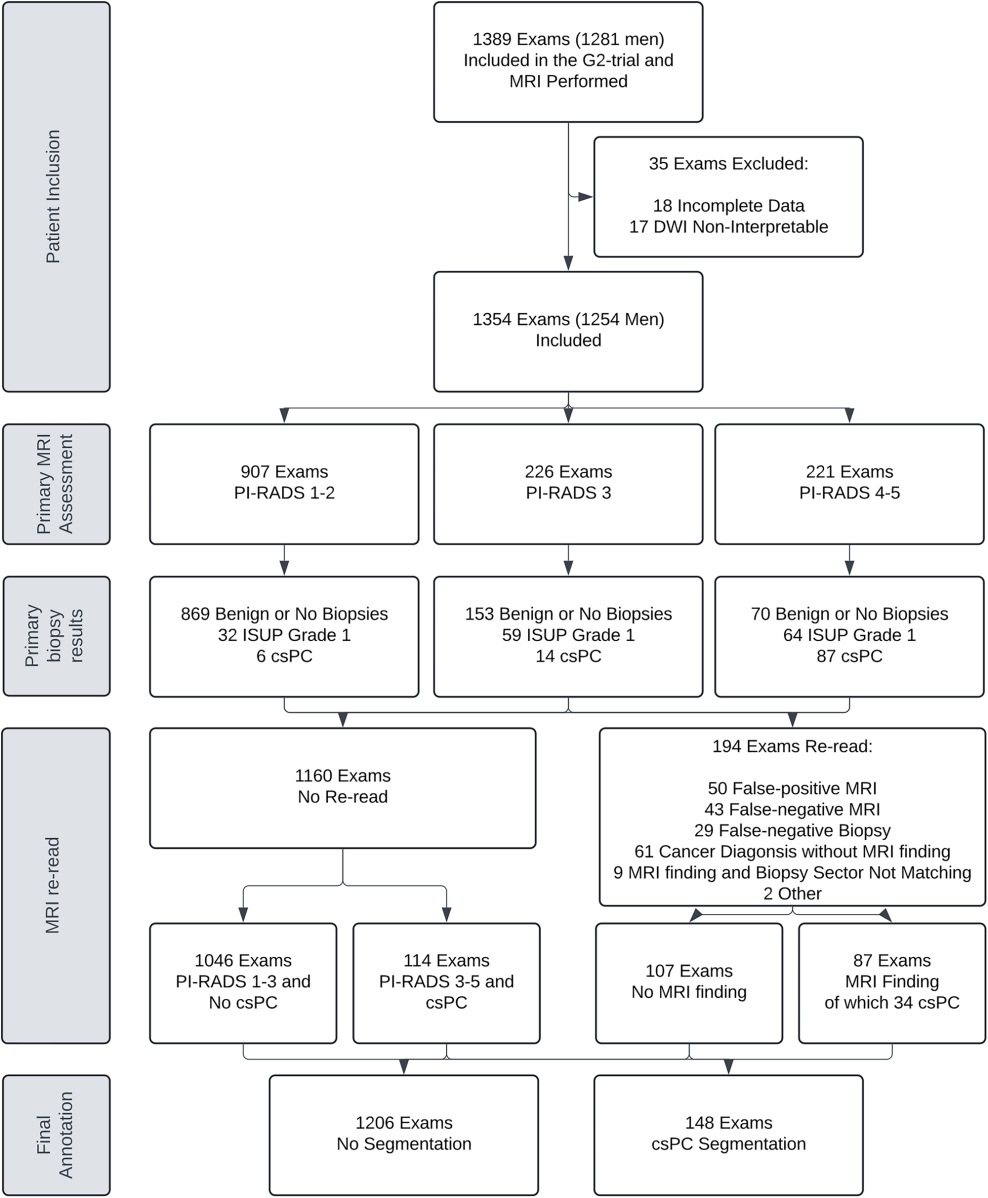
Flowchart of inclusion and determination of ground-truth in the final dataset. ISUP, International Society of Uropathology; csPC, clinically significant prostate cancer

Within the specified period, 1389 MRI examinations were performed (see Fig. 2). Of these, 35 examinations were excluded, either because of incomplete or missing data (*n* = 18) or because the DWI data were deemed non-interpretable due to hip prosthesis (*n* = 17). A total of 1354 examinations were included (see Table 1 for details about the training and test dataset). The median follow-up time for each MRI was 5.03 years (range 3.90–7.25 years).

**Table 1:** Included data

Dataset	Training	Test	Total
No. of exams	1086	268	1354
No. of men	1003	251	1254
Median age at MRI (years)	58	58	58
Mean PSA (ng/mL)	4.44	4.41	4.43
Primary MRI assessment (bpMRI score)			
PI-RADS 1–2	722 (66.5%)	185 (69%)	907 (67%)
PI-RADS 3	184 (16.9%)	42 (15.7%)	226 (16.7%)
PI-RADS 4	140 (12.9%)	25 (9.3%)	165 (12.2%)
PI-RADS 5	40 (3.7%)	16 (6.0%)	56 (4.1%)
Systematic biopsies performed	232	56	288
PI-RADS 1–2 and csPC	5	0	5
Re-read: False-negative MRI	3	0	3
Re-read: No MRI visible lesion*	2	0	2
No csPC in screening but csPC during follow-up	23	4	27
Re-read: False-negative MRI	11	2	13
Re-read: False-negative biopsy	6	0	6
Re-read: No MRI visible lesion*	5	3	8
PI-RADS 4–5 and no csPC during follow-up	90	19	109
ISUP grade 1	54	8	62
Re-read: False-positive MRI	25	7	32
Re-read: False-negative biopsy**	11	4	15
Reference			
CsPC not present	967 (89.0%)	239 (89.2%)	1206 (89.1%)
CsPC present	119 (11.0%)	29 (10.8%)	148 (10.9%)

* Classified as “csPC not present” despite biopsy showing csPC

** Classified as “csPC present” despite no histological verification

### Performance of AI system

**Fig. 3:**
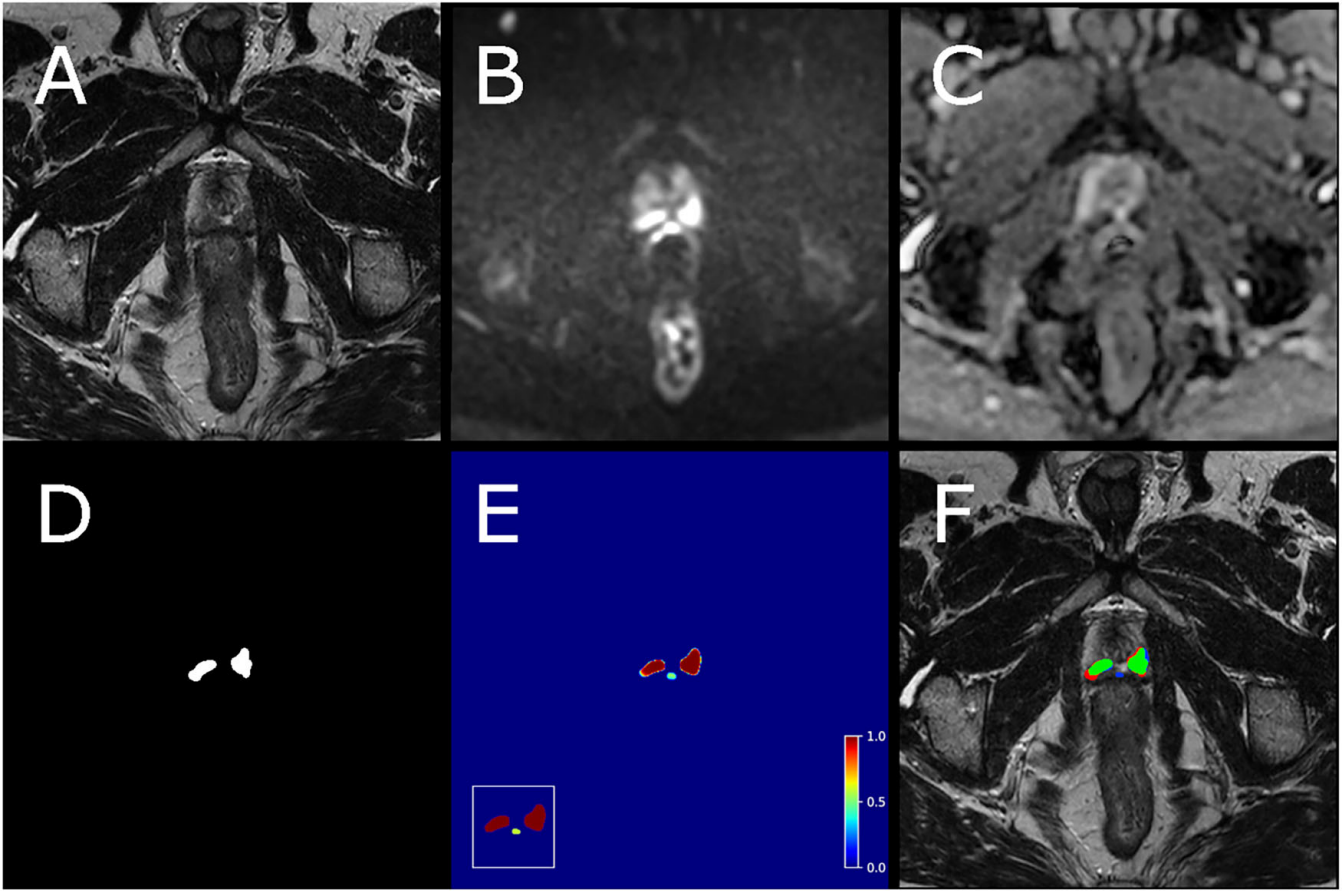
Example case from test set with biparametric MRI data, reference segmentation and output from AI system. The primary MRI assessment resulted in a PI-RADS 5 lesion in the left dorsal part of the peripheral zone and a PI-RADS 4 lesion in the right dorsal part of the peripheral zone (PSA of 11.6 µg/L). Targeted biopsies from both areas showed ISUP-grade 3. All images are from the same slice location showing a part of both lesions. **A** T2-weighted (T2W) image. **B** Diffusion-weighted image with a b-value of 1500 s/mm^2^. **C** Apparent diffusion coefficient map. **D** Reference segmentation with white areas representing tumor. **E** Softmax output from AI system. Inset in the lower left corner shows the detection map (cropped around the prostate) with red voxels representing softmax values of 0.99 and yellow voxels representing softmax values of 0.59. **F** T2W image with reference (**D**) and detection map (inset in **E**) overlayed, green represents areas in which both the reference and the AI output are positive (true positive voxels for AI), red only positive in reference (false-negative voxels for AI) and blue only segmented by AI (false-positive voxels for AI)

**Fig. 4:**
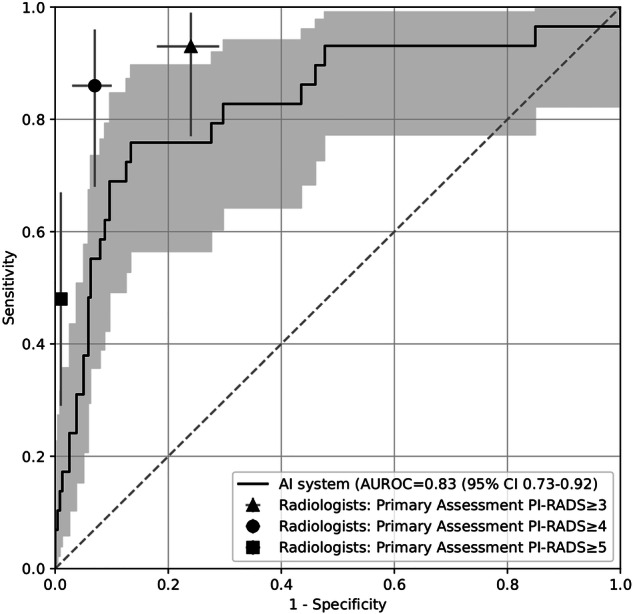
Receiver operating characteristic (ROC) curve for the AI system (with 95% confidence interval in gray) compared to the specificity and the sensitivity for the radiologists’ primary assessment using PI-RADS v2 (with 95% confidence interval). Evaluated on the test dataset (*n* = 268). AUROC, area under the receiver operating curve

**Fig. 5:**
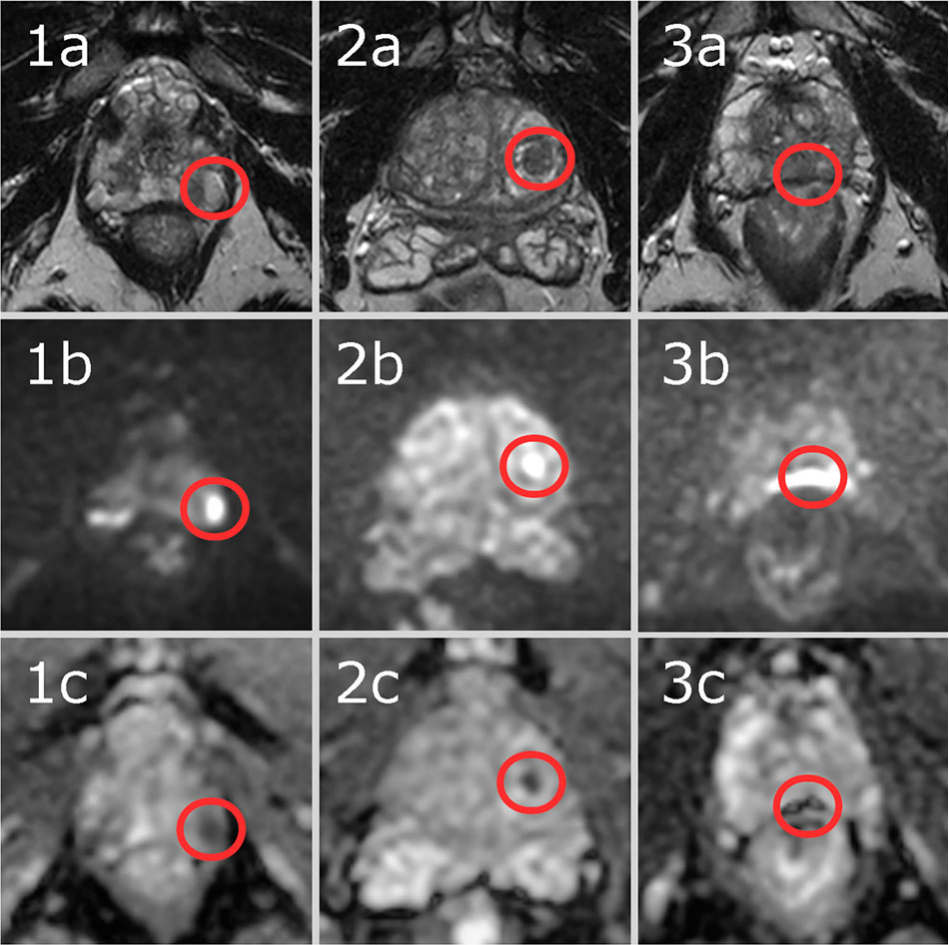
Three example cases (case 1, 2 and 3) from the test set with a negative reference (clinically significant prostate cancer not present), false-positive outputs by the AI system and true negative primary MRI assessment by radiologists. These three cases were selected to represent typical cases of false-positive AI outputs. For each case, a T2-weighted image (**a**), a high b-value image (**b**) and an apparent diffusion coefficient map (**c**) cropped around the prostate are shown from the same slice centered over the AI system’s output. The AI system’s findings are marked with a red circle surrounding the area instead of the segmentation, to improve visibility of the findings. Case 1. Man with PSA 3.0 ng/mL and a prostate volume of 33 mL resulting in a PSA-density of 0.09 ng/mL^2^. Systematic biopsies were performed and showed benign results. The AI system marked a lesion in the left apical part of the prostate. In the area, a periprostatic vein with markedly restricted diffusion can be seen. Case 2. Man with PSA 13.6 ng/mL and a prostate volume of 91 mL resulting in a PSA-density of 0.15 ng/mL^2^. No biopsies were performed. The AI system marked a lesion in the left transitional zone. In the area, a well-circumscribed lesion in the transitional zone can be seen with markedly restricted diffusion. Case 3. Man with PSA 2.3 ng/mL and a prostate volume of 30 mL resulting in a PSA-density of 0.08 ng/mL^2^. No biopsies were performed. The AI system marked a lesion in the middle dorsal part of the prostate. In the area, severe pile-up artifacts on the diffusion-weighted sequence caused by gas in the rectum can be seen

Training of both configurations (with the five folds trained simultaneously on separate GPUs) took 20 h and 23 min. Inference took approximately 30 s per case. In Fig. 3, an example case from the test set is shown, with the input data, including the reference segmentation and the output from the AI system. In the test set, the AI system produced an AUROC of 0.83 (95% CI 0.73–0.92). The ROC, including a comparison with the radiologists’ biparametric score from the primary MRI evaluation, is presented in Fig. 4. Specificities of the AI system and the radiologists at fixed sensitivity levels matching the radiologists’ sensitivity at PI-RADS 3, 4, and 5, along with confidence intervals for both, are shown in Table 2. An analysis of the false positives by the AI system is provided in Table 3, and three examples of false-positive cases are shown in Fig. 5.

**Table 2:** Specificity (with confidence interval) of the radiologists and the AI at fixed sensitivity levels, matching the radiologists’ primary assessment at PI-RADS score 3, 4 and 5

Primary assessment	Sensitivity	Specificity Radiologists (CI)	Specificity AI system (CI)	Specificity Radiologists – AI system (CI)
PI-RADS 3	0.93	0.76 (0.71–0.82)	0.52 (0.46–0.59)	0.24 (0.16–0.31)
PI-RADS 4	0.86	0.93 (0.90–0.96)	0.56 (0.50–0.63)	0.37 (0.30–0.43)
PI-RADS 5	0.48	0.99 (0.98–1.00)	0.94 (0.91–0.97)	0.05 (0.02–0.08)

*CI* 95% confidence interval calculated using bootstrapping

**Table 3:** AI system’s false positives in the test set

Total cases	268
AI system positive*	141
AI system true positive	27
AI system false positive	114
Primary MRI assessment PI-RADS 1–2	81
Not subject to biopsies	66
ISUP grade 1	4
Benign biopsies	11
Primary MRI assessment PI-RADS 3	21
Primary MRI assessment PI-RADS 4	10
Primary MRI assessment PI-RADS 5	2

* Threshold selected to match the sensitivity of primary MRI assessment by radiologists PI-RADS ≥ 3 (0.93)

## Discussion

A curated and annotated prostate MRI dataset from a screening population was prepared and used to train and evaluate a deep-learning-based segmentation method (nnU-Net) for the detection of csPC. The AI system achieved an AUROC of 0.83 (95% CI 0.73–0.92) in the test set. At the same sensitivity as the radiologists’ PI-RADS 3, 4, and 5, the AI system resulted in a lower specificity compared to the radiologists.

To our knowledge, no previous studies of the same scale exist on the training and evaluation of neural networks for prostate cancer detection in MRI data from a screening population. A small study using a similar dataset with 49 cases was published in 2020 [21] and another one with 57 cases in 2024 [22]. The employed screening dataset (*n* = 1354) has a prevalence of csPC of 10% and a mean PSA of 4.43 ng/mL. The previously published PI-CAI dataset (*n* = 10,207) has a prevalence of csPC of 24% and a median PSA of 8 ng/mL [13]. This indicates significant differences in csPC prevalence between screening and clinical populations. Accordingly, a cautious comparison of AI performance across different populations should be made. In the PI-CAI study, the ensemble of the five best algorithms in their challenge achieved an AUROC of 0.91 (CI 0.87–0.94) in their test set of 400 examinations [13]. This is slightly higher than the AUROC achieved by the present AI system, although it falls within the 95% CI. There may be several factors that explain this difference: the larger training dataset (9207 cases vs 1086 cases), better algorithms, and the more challenging nature of a screening dataset. The latter may indicate that AI systems at least have to be validated in a screening population to ensure optimal performance. The radiologists in the same study (using a 0 to 100 probability score and not PI-RADS) achieved an AUROC of 0.86 (CI 0.83–0.89). A 2020 meta-analysis of machine learning applications for the detection of csPC showed a pooled AUROC of 0.86 (CI 0.81–0.91) [23]. Commercially available systems with either CE marking or FDA approval evaluated with similar methodologies have demonstrated AUCs ranging from 0.79 to 0.91, although the datasets had much higher csPC prevalence (31–69%) compared with the present study [24–27]. There are many potential benefits of using AI for MR image interpretation in a screening setting but also potential pitfalls. The main advantage of using MRI before biopsies in prostate cancer screening is the reduction of overdiagnosis [28–30]. This effect might be reduced if the MRI-derived diagnosis yields too many false positives. Positives would also have to either be dismissed by a radiologist as false positive or be verified with biopsies in order to rule out csPC. This would result in an increase of the number of men who are subject to biopsies, which in turn creates an additional risk for the men. At the radiologists’ PI-RADS 3 sensitivity level (sensitivity = 0.96), the AI system used in this study resulted in a lower specificity compared to radiologists (mean difference 0.24, CI 0.16–0.31), which means that the AI system produces more false-positive findings. For lower sensitivity, the difference was smaller but still significant. In many of the false-positive cases, the AI segmented an area with markedly restricted diffusion. While this is a sign of malignancy, it can occur for other reasons. In the examples provided in Fig. 5, three typical cases are shown with false positives resulting from a periprostatic vein (case 1), an atypical nodule in the transitional zone (case 2), and a susceptibility artifact caused by rectal gas (case 3). In all of these cases, the location of the finding is important, something that the AI system seems to have failed to take into account. In the PI-CAI study, a comparison of specificity for AI systems and radiologists in multidisciplinary practice (using PI-RADS) at matched sensitivity level for PI-RADS 3 showed no significant difference (mean difference −0.002 (CI −0.038 to 0.035)). The lower specificity may have an impact on the clinical value of the MRI examination. The authors would like to caution readers to carefully validate the performance of AI systems when employing such systems in a screening setting, in order to at least understand the extent of false positives. It should also be noted that there are several potential diagnostic applications for AI systems in screening, for example, as a double reader to radiologists, which has proven useful in breast cancer screening [31] or, considering the low prevalence of csPC in prostate cancer screening, an automatic rule-out system seems advantageous [32]. Depending on the application, AI systems may have to be tweaked to ensure optimal performance, for example, to maximize sensitivity in a rule-out system.

The present study has some limitations. First, the dataset only includes examinations from a screening population with MRI examinations performed on the same MRI scanner using the same protocol, which, in theory, significantly limits generalization to other populations and scanners. On the other hand, the performance in this homogeneous setting might be superior compared to an AI system trained on more heterogeneous data. No evaluation of performance on external data was performed. To better assess generalizability, future studies should include external data from different scanners and hospitals. Second, the training and inference were performed using the segmentation method nn-UNet with default settings. While nn-UNet performs well out-of-the-box in csPC detection, as seen in the PI-CAI challenge [13], where two out of the top five networks were based on nnU-Net, as well as with other datasets [33]. Better performance might still be achieved with customization of the training and inference procedure. Third, some cases lack histological verification. A suitable reference standard is crucial when training and evaluating a neural network. The diagnostic workflow for prostate cancer is today primarily based on MRI findings, and European guidelines do not recommend systematic biopsies in MRI-negative men [1]. When curating a dataset, this causes problems since an MRI-negative case often lacks histological verification. Two methods were used in the present study to minimize the bias created by the lack of verification of MRI-negative cases: systematic biopsies in a subset of patients regardless of MRI findings and follow-up for at least 3 years. Also, MRI cases with high likelihood of csPC (PI-RADS 4–5) but no finding of csPC in targeted biopsies also pose challenges. There are several possible explanations for such a scenario; one is a false-negative biopsy [34]. To catch these cases, a re-read was performed, and 15 cases were included as csPC despite having no confirmed histological diagnosis. This approach introduces a bias in favor of the radiologists during evaluation, since the radiologists, in some cases, decide the reference. This must be considered when interpreting the results. However, during training, this approach seems advantageous since the AI can be trained to better find the clinically significant prostate cancer cases. For a completely non-biased evaluation, a prospective, randomized trial should be conducted. Fourth, in the present study, biparametric MRI data were used for training and evaluation of the AI system, omitting the DCE sequence. A biparametric protocol appears advantageous in a screening scenario, resulting in shorter examination time, lower cost, and no potential harm due to gadolinium contrast agents while maintaining a similar detection rate and producing fewer false positives [28, 30, 35]. Moreover, omitting the DCE results in much less data to be handled by the AI system, reducing data transfer and computation times. Notably, the radiologists had access to the multiparametric data at the time of primary assessment, which may give them an advantage over the AI system, but the reported PI-RADS scores were based on biparametric data. Fifth, the ADC map was calculated using four *b*-values (0, 100, 1000 and 1500 s/mm^2^), which is non-compliant with the PI-RADS recommendations. However, this particular choice of b-values has been investigated before, and the effects on lesion contrast on ADC maps can be considered negligible [36]. Alternative protocol approaches for consistent computation of the ADC could be considered [37].

To conclude, a prostate MRI dataset was curated from a screening population with histological confirmation of most positive cases and verification of a subset of negative cases using systematic biopsies and a median follow-up of 5 years. A neural network trained on this dataset produced lower specificities than the radiologists.

## Supplementary information

ELECTRONIC SUPPLEMENTARY MATERIAL

## Abbreviations

ADC: Apparent diffusion coefficient
AI: Artificial intelligence
AUROC: Area under the receiver operating characteristic curve
csPC: Clinically significant prostate cancer
DWI: Diffusion-weighted imaging
G2-trial: Göteborg prostate cancer screening 2 trial
ISUP: International Society of Urological Pathology
PC: Prostate cancer
PI-RADS: Prostate Imaging Reporting and Data System
PSA: Prostate-specific antigen
RGA: Report-guided annotation
ROC: Receiver operating characteristic
T2W: T2-weighted
