# Supplementary material for: Evaluation of AI for prostate cancer detection in biparametric-MRI screening population data
Source: Eur Radiol. 2025 Dec 8;36(5):3418–28. doi: 10.1007/s00330-025-12198-5 (PMC13086701; doi:10.1007/s00330-025-12198-5)
Supplement: Supplementary file 1 — ELECTRONIC SUPPLEMENTARY MATERIAL [file 330_2025_12198_MOESM1_ESM.pdf]

Evaluation of AI for Prostate Cancer Detection in Biparametric-MRI Screening Population Data

ELECTRONIC SUPPLEMENTARY MATERIAL

The scripts used in the manuscript is available at our public Github:

https://github.com/flangkilde/prostatemri_AI_eurorad

Please read the readme.md file for instructions.
